# Atherothrombosis is a Thrombotic, not Inflammatory Disease

**DOI:** 10.7759/cureus.1909

**Published:** 2017-12-05

**Authors:** Gregory D Sloop, Joseph J Weidman, John A St. Cyr

**Affiliations:** 1 N/A, Independent Researcher; 2 Research and Development, Jacqmar, Inc.

**Keywords:** blood viscosity, fatty streak, endothelial dysfunction, vulnerable plaque, obesity, atherothrombosis, thrombophilia, coagulopathy, metabolic syndrome, high sensitivity c-reactive protein

## Abstract

The authors hypothesize that thrombosis causes both the complications of atherosclerosis as well as the underlying lesion, the atherosclerotic plaque, which develops from the organization of mural thrombi. These form in areas of slow blood flow, which develop because of flow separation created by changing vascular geometry and elevated blood viscosity. Many phenomena typically ascribed to inflammation or “chronic oxidative stress”, such as the development of fatty streaks, “endothelial dysfunction,” “vulnerable plaques,” and the association of mild elevations of C-reactive protein and cytokines with atherothrombosis are better explained by hemorheologic and hemodynamic abnormalities, particularly elevated blood viscosity. Elevated blood viscosity decreases the perfusion of skeletal muscle, leading to myocyte expression of the myokine IL-6, decreased glucose uptake, insulin resistance, hyperglycemia, and metabolic syndrome. The hyperfibrinogenemia and hypergammaglobulinemia present in true inflammatory diseases foster atherothrombosis by increasing blood viscosity.

## Introduction and background

Recognizing the limited success of lipid-lowering therapy, atherothrombosis is now thought to be an inflammatory disease based largely on its association with slight elevations of C-reactive protein (CRP), an acute-phase reactant [1]. Detection of the slight elevations of CRP associated with an increased risk of cardiovascular disease, ≥ 2 mg/L, requires a “high-sensitivity” CRP assay, not the routine assay used to assess inflammation in other diseases. In contrast, the authors hypothesize that atherothrombosis is a thrombotic disease. The organization of mural thrombi results in an atherosclerotic plaque, while superimposed thrombosis leads to complications such as myocardial infarction and ischemic stroke [2]. In this paper, the authors will briefly review how hemodynamic and hemorheologic abnormalities can cause thrombosis. Furthermore, the authors review in depth the data offered to support an inflammatory etiology for atherothrombosis, including the Canakinumab Anti-Inflammatory Thrombosis Outcomes Study (CANTOS).

## Review

The hemorheologic and hemodynamic basis for atherothrombosis

Mural thrombi develop in areas of low shear stress created by flow separation caused by changing arterial geometry, such as branching, curving, dilatation, and obstructions like atherosclerotic plaques [2]. The formation of flow separation is fostered by arterial stiffening, which increases peak arterial velocity and the Reynolds number, which describes the likelihood of developing flow separation. Arterial stiffening progresses with aging and is accelerated in hypertension, male gender, and diabetes.

Because blood is a non-Newtonian fluid, blood viscosity increases in the areas of low shear stress created by flow separation. This increase in blood viscosity is augmented in hypercholesterolemia, hyperfibrinogenemia, hypergammaglobulinemia, hypertension, diabetes, aging, and several other risk factors for accelerated atherothrombosis. Increased blood viscosity creates larger areas of lower shear stress or slower flow, fostering atherothrombosis as well as superimposed thrombosis on atherosclerotic plaques. The propensity for thrombosis in areas of low shear is commonly attributed to the 19th century German pathologist Rudolph Virchow. Areas of low shear stress are predisposed to thrombosis because of decreased shear-mediated expression of molecules with anti-platelet activity such as nitric oxide and prostacyclin, decreased dispersion of activated coagulation factors, and decreased influx of fibrinolytic molecules [2]. Further, hematologic disorders which affect thrombosis, such as thrombophilias, bleeding dyscrasias, and hypofibrinolysis affect atherogenesis, not just superimposed thrombosis. Drugs that affect thrombosis such as aspirin, erythropoiesis-stimulating agents, and androgens also affect atherogenesis and superimposed thrombosis.

The idea that atherosclerotic plaques develop from the organization of mural thrombi was originally postulated by the Welsh pathologist John Duguid in the mid-twentieth century. Duguid’s theory remained popular until hypercholesterolemia was accepted as a risk factor for atherothrombosis and fatty streaks were hypothesized to be the precursor to atherosclerotic plaques. The discovery that low-density lipoprotein (LDL) increases blood viscosity and high-density lipoprotein (HDL) decreases blood viscosity [3] explains the impact of lipoproteins on atherothrombosis. In addition, the hemorheologic effects of hyperfibrinogenemia and hypergammaglobulinemia explain the increased risk of atherothrombosis noted in true inflammatory diseases.

A critical analysis of current views of inflammation

In recent years, chronic inflammation has been broadly, and some would say uncritically, associated with a panoply of conditions, including obesity and poorly understood chronic conditions such as metabolic syndrome, Alzheimer disease, psychosocial stress [4], and, surprisingly, watching television [5]. Since the first century A.D., inflammation has been defined clinically as “tumor, rubor, dolor, and calor,” i.e., swelling, redness, pain, and heat. Histopathologically, chronic inflammation is defined by the presence of lymphocytes, plasma cells, and macrophages, which are not present in obesity [vide infra], metabolic syndrome, or to a significant degree in typical atherosclerotic plaques. Inflammation of an artery, or “arteritis,” is a circumferential, concentric process, not eccentric as are atherosclerotic plaques. Alzheimer disease is a neurodegenerative disease in which proteins in the β-pleated sheet conformation accumulate within cells until these cells are no longer viable [6]. Inflammation is not a histopathologic feature [7]. However, inflammation is prominent in encephalitis. Psychosocial stress and television viewing have no histopathology of which the authors are aware.

The Origin of Elevated CRP and Cytokines in the Absence of Histopathologic Inflammation

Slight elevations of CRP are seen in several conditions that are not inflammatory, such as hypertension, consumption of the Western diet, and arterial stiffening [8]. Nevertheless, slight elevations of CRP have been offered as evidence that obesity, diabetes, and the metabolic syndrome are inflammatory conditions, even though they are not associated with histopathologic inflammation. Slight elevations of CRP, interleukin-6 (IL-6), and tumor necrosis factor-alpha (TNF-α) levels can be explained by elevated blood viscosity and decreased perfusion of skeletal muscle. 

The optimal function of any tissue requires adequate perfusion. Skeletal muscle is the largest organ in humans, comprising about 40% of body weight and receives 25% of cardiac output at rest. Skeletal muscle is also the primary site of glucose uptake in the postprandial state. Under euglycemic, hyperinsulinemic conditions, approximately 80% of glucose uptake occurs in skeletal muscle [9]. Perfusion is inversely proportional to vascular resistance, which is directly proportional to blood viscosity. Therefore, elevated blood viscosity reduces perfusion and glucose delivery to skeletal muscle. In 1991, Julius and colleagues proposed that reduced perfusion of skeletal muscle causes insulin resistance [10]. In 1998, Høieggen, et al. demonstrated that blood viscosity is elevated in patients with metabolic syndrome. They demonstrated an inverse relationship between glucose uptake and blood viscosity [11]. In a prospective randomized study in 2012, Houschyar and colleagues demonstrated that in subjects with metabolic syndrome, two rounds of therapeutic phlebotomy, which lowers blood viscosity, decreased plasma glucose by 12.5 mg/dL compared to 2 mg/dL in controls. Blood pressure decreased 18.3 mmHg in subjects and only 0.2 mmHg in controls [12]. Decreased blood pressure suggests that the intervention lowered blood viscosity because blood pressure is directly proportional to blood viscosity. 

A state that reduces perfusion and glucose uptake by myocytes will prolong postprandial hyperglycemia. Diabetic and hypertensive microangiopathy, as seen in metabolic syndrome [13], will further reduce perfusion, glucose delivery, and prolong postprandial hyperglycemia. In its role as a myokine, IL-6 is produced by myocytes in response to depletion of intracellular glycogen. After exercise, plasma levels of IL-6 can increase 100 times. This elevation of IL-6 is independent of levels of markers of myocyte damage such as myoglobin and creatine kinase [14]. We hypothesize that in the metabolic syndrome, reduced myocyte perfusion decreases intracellular glycogen and upregulates myocyte expression of IL-6. Elevated IL-6 upregulates the expression of CRP [15], in this situation a phenomenon best described as “pseudoinflammation” because CRP elevations associated with true inflammation are commonly much greater. IL-6 increases the production of insulin by increasing the secretion of glucagon-like peptide-1 from alpha cells in the pancreatic islets of Langerhans and intestinal L cells [16]. Insulin then increases the synthesis of TNF-α in adipocytes [17]. When infused into the femoral artery, TNF-α has been shown to increase IL-6 synthesis and glucose uptake [18]. Thus, decreased perfusion of skeletal muscle initiates a cascade in which concentrations of IL-6, insulin, and TNF-α are increased to maintain glycogen levels in myocytes.

Further increases in blood viscosity, as seen with aging [19] or consumption of the Western diet, or progressive microangiopathy will further reduce perfusion of skeletal muscle, which can influence the development of diabetes mellitus type 2. Thus, the slight elevation of CRP levels associated with an increased risk of cardiovascular disease is probably due to reduced perfusion of skeletal muscle caused by elevated blood viscosity, hypertension, and diabetes, all of which are risk factors for atherothrombosis. The precise effect of this slight elevation of CRP levels is unknown.

Elevated levels of IL-6, TNF-α, and CRP can result from responses to maintain homeostasis. Kushner and colleagues have argued against calling conditions “inflammatory” based on elevated cytokine or CRP levels alone. They wrote, “expression or activation of inflammation-associated cytokines and transcription factors is often taken as evidence of an inflammatory process. Such conclusions are treacherous, because these molecules are multifunctional, playing many other roles unrelated to inflammation. Similarly, minor elevations in CRP levels do not necessarily indicate or reflect inflammation” [8]. IL-6 is produced by many cell types in response to several different stimuli [20]. The participation of IL-6 in both glucose metabolism and inflammation with its production by both myocytes and macrophages is a good example of the multifunctionality of cytokines. Slight elevations of CRP and cytokine levels are too nonspecific to support a diagnosis of inflammation. Histopathologic findings should be the sine qua non for a diagnosis of inflammation. A diagnosis of “inflammation” approaches nonfalsifiablility if anatomical evidence is not required.

Dietary Concerns

Consumption of the Western diet, defined as increased consumption of red meat, processed meat, sweets, desserts, French fries, and high-fat dairy products, is associated with slight increases of CRP and IL-6 levels [21]. Furthermore, a high-fat diet increases blood viscosity. *Trans* and some saturated fats increase LDL levels, which elevates blood viscosity. However, the major effect of a high fat diet on blood viscosity is to decrease erythrocyte deformability by decreasing erythrocyte cell membrane fluidity [22]. When incorporated into the erythrocyte cell membrane, *trans* and long chain saturated fatty acids stiffen the membrane just like they solidify dietary fats. All elements of the metabolic syndrome, including obesity, elevated blood viscosity, hyperglycemia, insulin resistance, and hypertension can be a consequence of eating a Western diet.

In addition to elevating blood viscosity, decreased erythrocyte deformability also interferes with glucose uptake in skeletal muscle by impairing regulation of vascular tone. When deoxygenated, erythrocytes release adenosine triphosphate (ATP) which dilatates pre-capillary sphincters, increasing local perfusion [23]. This increases local blood flow to meet the metabolic needs of active tissue. Erythrocyte release of ATP is a function of deformability [24]. Thus, decreased erythrocyte deformability decreases ATP release and reduces local vasodilation. Decreased erythrocyte deformability is a common abnormality seen in hyperglycemia, hypercholesterolemia [25], some cases of hypertension, and in older erythrocytes.

Obesity

Adipose tissue from the obese is commonly examined histopathologically in specimens from breast reduction mammoplasties and panniculectomies. Furthermore, histopathologic examination of adipose tissue is performed daily in breast biopsies, deep skin biopsies, lymphadenectomies, colectomies, and omental biopsies, some of which are from the obese. If chronic inflammation was a manifestation of obesity in humans, it would have been identified.

The misconception that inflammation is a manifestation of obesity was supported by two reports which described lesions composed of multinucleate giant cells and lipid-containing macrophages surrounding necrotic adipocytes in genetically obese mice [26-27]. These lesions were not recognized to be a specific entity, i.e. fat necrosis. Instead, the investigators speculated that chronic inflammation is a spontaneous manifestation of obesity. In animals, fat necrosis occurs in the subcutis, abdomen, and retroperitoneum. In the subcutis, the lesion is thought to be due to trauma, while in the abdomen and retroperitoneum it is thought to be due to abnormal fatty acid metabolism [28]. Accentuated subcutaneous trauma is plausible in an unnaturally obese mouse. Humans with fat necrosis of the breast are usually corpulent with large, full breasts [29]. Abnormal fatty acid metabolism is also plausible in genetically obese mice in which fat storage genes are overexpressed. In humans, fat necrosis is a not-uncommon diagnosis in breast biopsies because it is difficult to distinguish from carcinoma by mammography. It is most often attributed to isolated trauma. In animals and humans, the lesion is a response to damaged or necrotic adipocytes. However, the error that inflammation is a manifestation of obesity continues to be perpetuated [30]. To their credit, the authors of both papers included photomicrographs, allowing proper diagnosis  retrospectively.

The overlap of inflammation and thrombosis in atherogenesis

Patients with chronic inflammatory diseases such as rheumatoid arthritis and systemic lupus erythematosus are at increased risk for atherothrombosis. Blood viscosity is increased in these patients because of hyperfibrinogenemia, hypergammaglobulinemia, immune complexes, and, to a lesser degree, other acute phase reactants. Blood viscosity correlates with disease activity following therapy in rheumatoid arthritis [31]. Successful treatment of active rheumatoid arthritis with the anti-inflammatory agent methotrexate will decrease blood viscosity and the subsequent risk of cardiovascular disease. Atherothrombosis associated with inflammatory disease is a special case because the associated elevated blood viscosity is not caused by the usual etiologies such as decreased erythrocyte deformability, increased hematocrit, dyslipidemia, etc.

In patients with type 2 diabetes mellitus or metabolic syndrome, neutralization of circulating TNF-α does not improve insulin sensitivity [18] because the elevated TNF-α levels are a homeostatic response to maintain cytoplasmic glycogen in myocytes, not driven by inflammation. In contrast, treatment of true inflammatory diseases with TNF-α-neutralizing therapy can improve insulin resistance [32] if the inflammation is severe enough to elevate blood viscosity. In both scenarios, the etiology of insulin resistance is elevated blood viscosity. However, only in the latter is the elevated blood viscosity (with its subsequent risk of atherothrombosis) treatable with immunomodulatory therapy.

The Canakinumab Anti-Inflammatory Thrombosis Outcomes Study (CANTOS) is purported to be a test of the inflammatory theory of atherothrombosis. In this study, subjects were randomized to receive either canakinumab, a monoclonal antibody directed against interleukin-1β, or placebo. Interleukin-1 is produced early in both the acute and chronic inflammatory responses. Subjects receiving 150 mg of canakinumab subcutaneously every three months had a significantly lower rate of recurrent cardiovascular events compared to placebo (95% confidence interval, 0.74 to 098, p=0.021) after a median follow-up of 3.7 years. Plasma CRP decreased but plasma lipid levels were unchanged. A Phase IIB trial of canakinumab revealed that treatment decreased plasma fibrinogen levels 21%, from 330 mg/dL to 260 mg/dL [33]. A meta-analysis of fibrinogen as a cardiovascular risk factor showed that even a modest (10%) increase in fibrinogen was associated with an increased risk of coronary artery disease [34]. Fibrinogen, like LDL, can foster erythrocyte aggregation and increase blood viscosity because its molecular diameter is large enough to simultaneously bind two erythrocytes. Fibrinogen also directly affects plasma viscosity. Thus, the beneficial effects of canakinumab could be due to decreased plasma fibrinogen concentrations and blood viscosity, not decreased inflammation. Unfortunately, fibrinogen concentrations were not reported in the Phase III trial. The Cardiovascular Inflammation Reduction Trial (CIRT) study, which will test the effect of methotrexate on recurrent myocardial infarction, is a more valid test of the inflammatory basis of atherothrombosis because low-dose methotrexate reportedly does not decrease plasma fibrinogen levels [35]. Presumably, this refers to the intrinsic activity of methotrexate, because successful treatment of inflammation with methotrexate will decrease the concentration of acute phase reactants, including fibrinogen.

An alternate view of the pathogenesis of atherothrombosis

Mainstream atherogenesis theory exploits putative phenomena such as “chronic oxidative stress” and “reverse transport of cholesterol” which were seemingly invented to explain the risk of atherothrombosis associated with LDL and HDL, respectively. It is much more likely that the pathophysiologic processes involved in atherogenesis are the same as those involved in homeostasis and other disease processes.

Fatty Streaks

Mainstream atherogenesis theory proposes that fatty streaks are the precursor to atherosclerotic plaques while acknowledging that, for unknown reasons, some fatty streaks spontaneously resolve without progressing. The accumulation of oxidized low-density lipoprotein (oxLDL) in the intima is felt to cause chemoattraction of monocytes, which mature into macrophages and phagocytize oxLDL via the scavenger receptor. OxLDL accumulates because of inadequate reverse transport of cholesterol by HDL. Ultimately, inflammation elicited by oxLDL causes fibrosis, which culminates in the formation of an atherosclerotic plaque. Foam cells, the characteristic histopathologic feature of fatty streaks, are held to be macrophages filled with cholesterol and cholesterol ester. Identical cells are present in the gall bladder mucosa in cholesterolosis, although that condition is non-inflammatory. Oxidative modification of LDL is felt to be necessary because uptake via the LDL receptor is subject to negative feedback and cannot result in sufficient accumulation of cholesterol to form foam cells. This has led to the concept of “chronic oxidative stress.”

The characteristic patterns of aortic fatty streak evolve between ages 15 and 42 because of progressive vascular stiffening, which decreases retrograde blood flow during diastole. Unsteady blood flow caused by the interaction of antegrade and retrograde blood flow in late systole/early diastole creates gaps in the underlying endothelium [36], impairing its barrier function which increases the movement of plasma constituents into the subendothelial space. There, dendritic cells, a normal component of the subendothelium [37], constitutively sample the aqueous environment by fluid-phase macropinocytosis [38] [vide infra] as part of their immunologic sentinel function. Dendritic cells, also known as fixed or tissue macrophages, are distinguished from the macrophages present in chronic inflammation by their distinctive ruffled cell membrane, which maximizes their surface area and environmental contact. Consequently, dendritic cells can pinocytose 100x their volume per hour in vitro [39], accumulating all plasma macromolecules, including LDL. Macropinocytosis is not receptor-dependent, making oxidative modification unnecessary for the development of fatty streaks. In fact, immunohistology reveals that oxLDL does not localize to atherosclerotic lesions, is not associated with inflammation, and is found in coronary veins, which do not develop atherosclerosis [40]. Dendritic cells develop from precursors in the embryonic yolk sac, not bone marrow-derived monocytes. In steady state conditions, monocytes do not substantially contribute to the formation of tissue macrophages, which are long-lived and self-renewing [41-42]. Thus, monocyte chemotaxis plays no significant role in the development of fatty streaks.

In macropinocytosis, the cell membrane engulfs the surrounding fluid and invaginates, forming vesicles called endosomes which subsequently fuse with lysosomes. Lysosomes lack enzymes capable of catabolizing cholesterol resulting in the accumulation of cholesterol-laden endosomes, forming a foam cell. Progressive aortic stiffening with age results in the migration of areas of unsteady blood flow and impaired endothelial barrier. The barrier function of previously affected areas normalizes, allowing the underlying fatty streak to resolve without sequelae [36]. Previous studies of foam cells in the gall bladder have revealed that their large size and rigidity hinders their ability to pass through lymphatic capillaries [43]. Instead, cholesterol and cholesterol ester are eliminated by exocytosis in membrane-bound vesicles called exosomes [44]. Exosomes form by inward budding of the endosome membrane creating multivesicular endosomes (MVE). The MVE then fuses with the cell membrane, releasing exosomes into the interstitial fluid. Exosomes circulate until they are cleared by the reticuloendothelial cells of the liver [45], which allows hepatocytes to break down the cholesterol into bile. Elimination of cholesterol via exosomes has been demonstrated in Niemann-Pick type C disease, a lipid storage disorder [46]. Therefore, cholesterol accumulation and elimination from dendritic cells occur by normal physiologic processes. It should not be surprising that a mechanism exists to handle molecules which have crossed the endothelium, which is so common as to be practically physiologic. Fatty streaks are nearly universal in childhood and adolescence regardless of the prevalence of atherosclerotic plaques in adults. The formation of foam cells suggests that the great capacity of dendritic cells for macropinocytosis allows cholesterol to accumulate faster than it can be eliminated by MVE formation and exocytosis. Inflammation, monocyte chemotaxis, oxidative stress, and reverse transport of cholesterol by HDL do not play a significant role in the development or resolution of fatty streaks.

Endothelial Dysfunction

The endothelium produces molecules with anti-thrombotic activity, such as nitric oxide and prostacyclin, in response to shear stress [47]. Deficient production of these molecules or “endothelial dysfunction” is widely felt to be a manifestation of chronic inflammation or oxidative stress. However, slow blood flow downregulates their production and increases the risk of thrombosis. Areas of slow blood flow develop naturally in arteries due to flow separation caused by changing arterial geometry. Because of these alterations, these sites are common locations for atherothrombosis [2]. Forward blood flow is necessary to prevent apoptosis of endothelial cells [48]. Thus, endothelial cell loss will occur in areas of stasis, leading to superficial erosion with superimposed thrombosis. Nitric oxide causes relaxation of the smooth muscle in the tunica media following diffusion from the endothelium. Loss of nitric oxide will cause vasospasm, possibly leading to the rupture of the overlying plaque and superimposed thrombosis.

“Vulnerable” Atherosclerotic Plaques

An atherosclerotic plaque creates a partial obstruction which increases the velocity of blood flow across it, creating flow separation distally [49]. This area of flow separation is especially vulnerable to thrombosis because the rapid blood flow increases shear-mediated platelet activation. The atherosclerotic plaques which form there are more likely to develop superimposed thrombosis because of these adverse hemodynamics, resulting in what is commonly referred to as a “vulnerable plaque.” These plaques may become symptomatic earlier in the process of organization than proximal plaques. Thus, they may exhibit a thin fibrous cap, ample lipid and prominent neovascularization, which are the physical characteristics of a vulnerable plaque [2]. As with endothelial dysfunction, abnormal hemodynamics contributes to the development of vulnerable plaques, not a state of chronic inflammation or oxidative stress.

## Conclusions

Atherothrombosis is a thrombotic, not inflammatory disease. On a practical level, a change in the focus of atherothrombosis research from inflammation and lipid metabolism to thrombosis is desirable given that risk factors such as age and hematocrit have no obvious association with either inflammation or dyslipidemia. The origin of atherosclerotic plaques from the organization of mural thrombi explains why varied risk factors all result in the same morphologic lesion, the atherosclerotic plaque. Mainstream atherothrombosis theory sheds no light on how hypertension, male gender, thrombophilias, hypofibrinolysis, etc. accelerate the development of atherosclerotic plaques. Because an elevated LDL concentration is only one of several causes of increased blood viscosity, interventions which decrease LDL levels have limited efficacy in preventing atherothrombosis. DuBroff cataloged 44 randomized controlled trials of cholesterol-lowering therapy which showed no benefit in cardiovascular mortality. Thirty of these trials showed no decrease in nonfatal cardiovascular events [50]. In addition to spurring the development of more effective therapies, insight into the true cause of atherothrombosis will inform nutritional recommendations. At present, there is great controversy regarding optimal fat intake, especially regarding saturated fats. Mainstream thought and billions of dollars spent on studies and drug development have given us mildly effective therapies and controversy regarding what we should eat. It is time for a paradigm shift.
